# Lymphatic malformations in children: treatment outcomes of sclerotherapy in a large cohort

**DOI:** 10.1007/s00431-020-03811-4

**Published:** 2020-10-13

**Authors:** Frédérique C. M. Bouwman, Silje S. Kooijman, Bas H. Verhoeven, Leo J. Schultze Kool, Carine J. M. van der Vleuten, Sanne M. B. I. Botden, Ivo de Blaauw

**Affiliations:** 1 Department of Surgery, Division of Pediatric Surgery, Radboudumc-Amalia Children’s Hospital, Nijmegen, Netherlands; 2 Department of Radiology and Nuclear Medicine, Division of Interventional Radiology, Radboud University Medical Center (Radboudumc), Nijmegen, Netherlands; 3 Department of Dermatology, Radboud University Medical Center (Radboudumc), Nijmegen, Netherlands; 4 Center for Vascular Anomalies, Hecovan, Radboud University Medical Center (Radboudumc), VASCERN VASCA European Reference Center, Nijmegen, Netherlands

**Keywords:** Lymphatic malformation, Vascular malformation, Sclerotherapy, Bleomycin, Lauromacrogol

## Abstract

This retrospective study examines the outcomes of sclerotherapy in children with (veno)lymphatic malformations who received sclerotherapy between 2011 and 2016 (116 children, 234 procedures). Complication severity was classified using the Society of Interventional Radiology classification. Clinical response was rated on a scale of 0 (no change) to 3 (good improvement). The sclerosants used were bleomycin (*n* = 132; 56%), lauromacrogol (*n* = 42; 18%), doxycycline (*n* = 15; 6%), ethanol (*n* = 12; 5%), or a combination (*n* = 33; 14%). Four major and 25 minor complications occurred without significant differences between the agents. The median response rate per procedure was 2—some improvement—for all sclerosants. However, in pure LMs (67%), bleomycin and a combination of agents resulted in the best clinical response. On patient level, all had some or good clinical response. Mixed macrocystic and microcystic lesions showed a significantly lower clinical response (median 2 versus 3; *p* = 0.023 and *p* = 0.036, respectively) and required significantly more procedures (median 2 versus 1; *p* = 0.043 and *p* = 0.044, respectively) compared with lesions with one component.

*Conclusion*: Sclerotherapy for (V)LMs in children is safe and effective. Bleomycin is the most frequently used agent in this clinic and seemed most effective for pure LMs. Mixed macrocystic and microcystic lesions are most difficult to treat effectively.

**Table:** 

**What is Known:** • *A variety of agents can be used for sclerotherapy of lymphatic malformations in children.* • *Macrocystic lesions have favorable outcomes compared with microcystic and mixed lesions.*	
**What is New:** • *Bleomycin and a combination of agents seem to be most effective to treat lymphatic malformations in children.* • *Mixed macrocystic and microcystic lesions are more difficult to treat effectively compared with lesions with either one of these components.*

**What is Known:**

• *A variety of agents can be used for sclerotherapy of lymphatic malformations in children.*

• *Macrocystic lesions have favorable outcomes compared with microcystic and mixed lesions.*

**What is New:**

• *Bleomycin and a combination of agents seem to be most effective to treat lymphatic malformations in children.*

• *Mixed macrocystic and microcystic lesions are more difficult to treat effectively compared with lesions with either one of these components.*

## Introduction

Lymphatic malformations (LMs) are congenital developmental anomalies of the lymphatic system, with or without a venous component (venolymphatic malformation; VLM) [1]. The incidence is estimated to be 1 in 2000–6000 live births [2–5]. Symptoms, often named lesion-related complications, vary from pain and swelling to relapsing periods of infection and distortion of anatomical structures. Sclerotherapy is currently the gold standard for (V)LM treatment if invasive management is indicated [6], and a wide range of sclerosing agents have been used in daily practice, including bleomycin, lauromacrogol, doxycycline, ethanol, and picibanil.

Several studies evaluated treatment outcomes after use of various agents for sclerotherapy in (V)LMs [7–17]. However, most of these studies included small patient cohorts [9, 16, 17] or both adults and children [8, 11, 12, 15], which could confound or bias the results. Among these studies, those examining larger pediatric cohorts lack information on bleomycin [10, 13, 14], which is currently one of the most frequently used agents worldwide. In a systematic review on agents used for sclerotherapy, it is concluded that the optimal sclerotherapy could not be identified and that the difference in complication rates could be the deciding factor in the choice between agents [18].

Therefore, this study evaluates sclerotherapy outcomes of (V)LMs in children treated in a high volume center where different sclerosing agents are being used. These data will provide more insight into the optimal agent for sclerotherapy of (V)LMs and will provide guidance in the counseling of children and their parents on the treatment and prognosis.

## Methods

### Data collection

A retrospective analysis was performed of all children (< 18 years of age) with (V)LMs who received percutaneous sclerotherapy between January 2011 and December 2016. Minimal follow-up was 6 months. All patients were treated at the expertise center for hemangiomas and congenital vascular malformations (HECOVAN) at the Radboudumc-Amalia Children’s Hospital in Nijmegen, Netherlands. The local ethical committee approved this study.

Medical records were reviewed for patient age at diagnosis and procedure, sex, comorbidity, the number of sclerotherapy procedures during the study period, and the lesion-related complications prior to sclerotherapy. Lesion characteristics included anatomical location and morphological subtype. As regards the anatomical location, six regions were defined: (1) head/neck area, (2) thorax, (3) abdomen/pelvis, (4) upper extremity, (5) lower extremity, and (6) abdomen/pelvis and lower extremity. Morphologically, the lesions were classified as macrocystic, microcystic, or mixed macro- and microcystic and, secondly, as pure lymphatic (LM) or venolymphatic (VLM). The difference between macrocystic and microcystic was defined by the size and accessibility of the cysts [1, 6, 8, 19]. Macrocystic lesions contain cysts ≥ 2 cm or considered accessible for aspiration based on ultrasound. Microcystic lesions contain cysts < 2 cm or considered inaccessible for aspiration. Mixed lesions contain both macrocystic and microcystic components. VLMs have both venous and lymphatic components.

### Outcome parameters

The outcome parameters were short-term (< 30 days) and long-term procedure-related complications and clinical response rate. Post-treatment pain, local swelling, and transient fever without focus of infection were not considered complications, but expected side effects of sclerotherapy. The procedure-related complications were classified as minor or major according to the Society of Interventional Radiology (SIR) Classification System for Complications by Outcome (Table 1) [20]. Nominal therapy included oral antibiotic treatment. Complications requiring intravenous antibiotic treatment and thus hospitalization were classified as major.

**Table 1:** SIR Classification system for complications by outcome [20]

Minor complications	
A: No therapy; no consequence	
B: Nominal therapy; no consequence (includes overnight admission for observation only)	
Major complications	
C: Require treatment; minor hospitalization (< 48 h)	
D: Require major therapy, unplanned increase in level of care, prolonged hospitalization (> 48 h)	
E: Permanent adverse sequelae	
F: Death	

The clinical response was based on documentation in the patient records, similar to other studies [8, 10], and expressed on a scale of zero to three. Zero represents no response to treatment. One represents slight improvement, defined as little regression compared with the original size with almost no cosmetic relief or functional improvement. Two represents some improvement, defined as visible regression of the lesion compared with the original size with some cosmetic relief or functional improvement. Three represents a good response, defined as total or near-total volume reduction of the lesion with no or minimal residual cosmetic or functional impairment.

### Technique of sclerotherapy

Sclerotherapy is performed under general anesthesia. Sclerotherapy of lymphatic malformations in children can be challenging due to the small size of the cysts or lymph vessels and the limited safety margins of the agents used. In addition, some of the agents could generate an intense pain response. General anesthesia generates a controlled environment, facilitating targeted sclerotherapy: limiting the number of injections and radiation hazard needed and injecting the sclerosing agent as specific as possible. In our opinion, sclerotherapy under general anesthesia also minimizes psychic trauma. This is an important part to take into account, in particular because a treatment course of sclerotherapy could include multiple sessions.

After puncturing the lesion with a 21–23 gauge needle using ultrasound guidance, as much fluid as possible is aspirated. Either bleomycin (Bleomedac, Lamepro BV, Breda, Netherlands; 600 IU/kg, age < 1 year: max 10.000 IU, age > 1 year: max 15.000 IU per session), lauromacrogol (Aethoxysklerol, Chemical Factory Kreussler & Co., Wiesbaden, Germany; either as a liquid with a concentration of 2% or as foam; 2 ml lauromacrogol 2% with 8 ml sterile room air, max 20–30 ml foam), doxycycline (Vibramycin, Pfizer, Capelle aan den IJssel, Netherlands; 10 mg/ml, max 20 mg/kg), or ethanol (Alcohol, Pharmacy A15, Gorinchem, Netherlands; max 0.14 ml/kg/10 minutes and max 0.5–1.0 ml/kg per session) is injected under ultrasound guidance. Ethanol sclerotherapy for macrocystic lesions involves both injection and subsequent aspiration of the agent (rinsing). Additional fluoroscopy guidance is used in case of macrocystic lesions or a (suspected) venous component to visualize drainage into normal soft tissue or circulation.

### Choice of used agent

Bleomycin is the preferred sclerosant for LMs in our center and also one of the most frequently used agents worldwide [18]. The choice for another agent or a combination of agents was based on location, content, extent, morphological type of the (V)LM, and volume of bleomycin that could be used based on weight of the patient and the cumulative lifetime dose. At locations where only minimal swelling was allowed, such as the eyelid or in close proximity to a nerve, bleomycin was highly preferred over one of the other agents. In other cases, when swelling was allowed, for example, in case of an intra-abdominally located lesion, ethanol or lauromacrogol was used. Another aspect was the morphological subtype of the lesion. Microcystic lesions were preferably treated with bleomycin or doxycycline. The agent of choice for macrocystic lesions depended on the location as described previously. Venolymphatic malformations were often treated with ethanol or lauromacrogol in addition to bleomycin, because of the venous component. Ethanol was used preferentially in more severe or extensive lesions, and a combination of agents was often used in case of large lesions.

### Statistical analyses

Complications and response rates were compared on patient and procedure level between the different sclerosing agents, the regions of anatomical location, and the morphological subtypes. The statistical analyses were performed with IBM SPSS statistical software version 25. Categorical data were presented as frequencies and percentages and analyzed using Pearson’s chi-square or Fisher’s exact test where appropriate. For continuous data, means and standard deviations or medians and ranges were calculated depending on the distribution of data. For the results of clinical response (ordinal data), both means and medians were calculated. Analyses were performed using the independent *t* test or the ANOVA in case of normal distribution and the Mann-Whitney *U* test or Kruskal-Wallis test in case of non-normal distribution. A *p* value of < 0.05 was considered significant.

## Results

### Patient demographics and lesion characteristics

A total of 234 sclerotherapy procedures were performed in 116 children (53 males, 63 females) during the study period. Table 2 summarizes the lesion characteristics of this study population. A pure LM was diagnosed in 78 patients (67%) and a VLM in 38 patients (33%). Age at first sclerotherapy procedure ranged from 1 day to 9 years, with a median of 7 years. Four patients required ex-utero intrapartum therapy (EXIT) with direct endotracheal intubation because of a potentially airway-threatening LM. Five patients had a (V)LM in the context of lymphangiomatosis (*n* = 2) or Klippel-Trenaunay syndrome (*n* = 2).

**Table 2:** Lesion characteristics

	Total group (*n* = 116)
Diagnosis of the lesion	
In utero	15 (13%)
At birth	47 (41%)
< 1 year	12 (10%)
1–2 years	10 (9%)
> 2 years	30 (26%)
Type of malformation	
Pure lymphatic	78 (67%)
Venolymphatic	38 (33%)
Morphological subtype	
Macrocystic	57 (49%)
Microcystic	22 (19%)
Mixed	37 (32%)
Anatomical location	
Head and neck	57 (49%)
Thorax	20 (17%)
Abdomen and pelvis	14 (12%)
Upper extremity	8 (7%)
Lower extremity	10 (9%)
Abdomen/pelvis/lower extremity	7 (6%)
Lesion-related complications	
Increase in size	70 (60%)
Pain	50 (43%)
Functional impairment	37 (32%)
Cosmetically disturbing lesion	34 (29%)
Infection	13 (11%)
Bleeding	11 (9%)
Oppression of other structures	10 (9%)
Airway permeability threatened	6 (5%)

Most patients suffered from multiple lesion-related complications before the first procedure during the study period (Table 2). Sixteen patients (14%) required treatment for their lesion-related complications prior to the study period, including antibiotics (*n* = 7), tracheal cannulation (*n* = 2), cyst drainage because of compression (*n* = 2), and amputation of the lower leg (*n* = 1). One-third of the patients (*n* = 36) received treatment for their malformations prior to the study period, of whom nine (8%) had a surgical resection.

The median number of sclerotherapy procedures per patient during the study period was one (range 1–13). Of all patients, 55% received one and 18% two procedures. The sclerosing agents used were bleomycin (*n* = 132; 56%), lauromacrogol (*n* = 42; 18%), doxycycline (*n* = 15; 6%), ethanol (*n* = 12; 5%), or a combination of agents (*n* = 33; 14%). Combination therapy consisted of bleomycin and ethanol (*n* = 6), bleomycin and lauromacrogol (*n* = 19), or lauromacrogol and ethanol (*n* = 8).

### Procedure-related complications

A total of 25 minor and four major complications were identified in 24 patients. No known cases of pulmonary toxicity were seen during the study period. Table 3 shows the procedure-related complications in the total group and for each sclerosing agent used. No significant differences were found in the occurrence of complications between the agents and between the different morphological subtypes. Post-procedural pain was an expected outcome, although it was specifically reported in twelve patients, of which seven treated with bleomycin, two with doxycycline, and the remainder all with different combinations of sclerosants.

**Table 3:** Procedure-related complications

	Total Group(*n* = 234)	Bleomycin (*n* = 132)	Lauromacrogol (*n* = 42)	Doxycycline (*n* = 15)	Ethanol (*n* = 12)	Combination (*n* = 33)	*p* value
Short term	26 (11%)	15 (11%)	5 (12%)	2 (13%)	–	4 (12%)	0.792
Minor							
Hematoma	16	10	2	1	–	3	0.819
Other	4	2	2	-	–	–	0.493
Infection	2	1	-	-	–	1	0.653
Major							
Infection/abscess	2	1	–	1	–	–	0.150
Functional impairment	2	1	1	–	–	–	0.793
Long term	3 (1%)	3 (2%)	–	–	–	–	0.672
Minor							
Scarring	1	1	–	–	–	–	0.942
Hyperpigmentation	1	1	–	–	–	–	0.942
Lymphoedema	1	1	–	–	–	–	0.942

A total of 22 short-term minor complications were identified. Hematoma was the most common complication (*n* = 16). Two patients were treated with oral antibiotics for an infection. One of these patients had a tracheal cannula because of the LM and developed pneumonia 7 days after sclerotherapy with bleomycin. The four “other” complications were skin necrosis (*n* = 1; bleomycin), febrile convulsion (*n* = 1; bleomycin), blistering (*n* = 1; lauromacrogol), and stridor (*n* = 1; lauromacrogol).

Four patients developed a short-term major complication. Two patients were diagnosed with an abscessed LM cyst, for which antibiotic treatment and drainage of the cyst were indicated. Two patients suffered from severe post-procedural swelling with functional impairment. One of these developed an abnormal gait and the other a reduced vision to 15%. Both required a resection of the swollen LM to reduce the functional impairment.

Three long-term procedure-related complications occurred during the study period, all after treatment with bleomycin. These were scarring, hyperpigmentation, and lymphedema. The lymphedema was treated with compression therapy.

### Clinical response rate

The clinical response to sclerotherapy with the various agents per procedure is summarized in Table 4. A total of 214 procedures (91%) resulted in “some” (*n* = 160; 68%) or “good” (*n* = 54; 23%) clinical improvement. A response of 2 (some) or 3 (good) was reported after 93% of the procedures in case of bleomycin or lauromacrogol injection, 75% after ethanol, 73% after doxycycline, and 97% after injection of a combination of agents. Table 5 shows the post hoc analysis of the differences in response rates shown in Table 4. For the total group, bleomycin and a combination of agents were significantly better than doxycycline (means 2.2 and 2.1 versus 1.7, *p* = 0.010 and *p* = 0.030). In pure lymphatic lesions, bleomycin (mean 2.2) and a combination of agents (mean 2.3) were significantly better than both doxycycline (mean 1.7; *p* = 0.021 and *p* = 0.014) and ethanol (mean 1.4; *p* = 0.023 and *p* = 0.020). Lauromacrogol resulted in a mean response rate of 2.1; this was not significantly different compared with the other agents.

**Table 4:** Response rate per procedure

Response rate (scale 0–3)	Total Group	Bleomycin	Lauromacrogol	Doxycycline	Ethanol	Combination	*p* value
Total group	*n* = 234	*n* = 132	*n* = 42	*n* = 15	*n* = 12	*n* = 33	
Mean Median (range)	2.1 2 (0–3)	2.2 2 (0–3)	2.1 2 (0–3)	1.7 2 (0–3)	1.9 2 (0–3)	2.1 2 (0–3)	0.071
Pure lymphatic	*n* = 142	*n* = 98	*n* = 14	*n* = 10	*n* = 5	*n* = 15	
Mean Median (range)	2.1 2 (0–3)	2.2 2 (0–3)	2.0 2 (0–3)	1.7 2 (0–2)	1.4 2 (0–2)	2.3 2 (2–3)	*0.021*
Venolymphatic	*n* = 92	*n* = 34	*n* = 28	*n* = 5	*n* = 7	*n* = 18	
Mean median (range)	2.1 2 (0–3)	2.1 2 (0–3)	2.1 2 (1–3)	1.8 2 (1–3)	2.3 2 (1–3)	1.9 2 (0–3)	0.431

**Table 5:** Post hoc analysis of response rate per procedure

	Total group	Pure lymphatic
*p* value	0.071	*0.021*
Bleomycin vs doxycycline	*0.010*	*0.021*
Bleomycin vs ethanol	0.282	*0.023*
Combination vs doxycycline	*0.030*	*0.014*
Combination vs ethanol	0.488	*0.020*
Lauromacrogol vs doxycycline	0.061	0.641
Lauromacrogol vs ethanol	0.243	0.147

In the evaluation on patient level, at the end of the study period, 65 patients (56%) had shown some clinical response after a median of two procedures (range 1–13), and 51 patients (44%) had shown good clinical response after a median of one procedure (range 1–4). The clinical response per patient was significantly influenced by the morphological subtype of the lesion (*p* = 0.042) and the location of the lesion (*p* = 0.043). The clinical response was better in macrocystic (median 3, range 2–3) and microcystic lesions (median 3, range 2–3) compared with mixed lesions (median 2, range 2–3; *p* = 0.023 and *p* = 0.036, respectively). The number of procedures performed per patient during the study period was also significantly higher in patients with a mixed lesion (median 2, range 1–13) compared with patients with a macrocystic (median 1, range 1–7; *p* = 0.043) or microcystic lesion (median 1, range 1–5; *p* = 0.044). The median response in patients with a lesion in the head and neck area was significantly better (median 3) than in patients with a lesion in the abdomen and pelvis (median 2; *p* = 0.028) or in the upper extremity (median 2; *p* = 0.028). At the end of the study period, seven patients of this cohort received treatment with an mTOR inhibitor due to an insufficient response to sclerotherapy on the long term.

## Discussion

This study aimed to evaluate the treatment of (V)LMs in a large pediatric cohort and found that bleomycin and a combination of agents were superior in the treatment of pure LMs. Furthermore, the study indicates that lesions with both macrocystic and microcystic components are more difficult to treat than lesions with either one of these components.

This study shows that all sclerosing agents are effective with some or good clinical improvement after 91% of the procedures. On patient level, all patients had shown some or good clinical response at the end of the study period. This is a good outcome compared with the range described in the systematic review of Horbach et al. showing an overall response rate ranging from 67 to 100% per patient [18].

The present study indicates that bleomycin and a combination of agents result in a better clinical response compared with doxycycline and ethanol. However, caution must be applied with the interpretation of our data, because doxycycline and especially ethanol were used in specific cases in this cohort. Doxycycline was used, for example, when bleomycin was relatively contraindicated or not available. Ethanol is the preferred agent, based on expert opinion, for more severe or extensive lesions or lesions with a venous component. Unfortunately, these data only included a small number of patients.

On patient level, the response rates were significantly influenced by the morphological subtype: (V)LMs with both macrocystic and microcystic components were found to have lower response rates than lesions with either one of these components. The median number of procedures per patient was also higher in these mixed (V)LMs. It has been described previously that better results were achieved in macrocystic LMs compared with microcystic and/or mixed lesions [7, 9, 21–25]. However, in contrast to these studies, the present study shows that the results in microcystic lesions were similar to those in macrocystic lesions. Despite the good results for microcystic and macrocystic lesions, it is unexplained why mixed lesions responded inferiorly to sclerotherapy.

This study shows that sclerotherapy is a good first-line treatment for (V)LMs in case invasive management is necessary. However, the beneficial results of sclerotherapy do not always persist on the long term or patients suffer from residual disease [26], which was also the case in some patients in this cohort. The mTOR inhibitor sirolimus has been found to be effective in lymphatic malformations that were refractory to standard treatment [27, 28]. Current clinical trials further investigate the use of sirolimus and which patients would benefit most from this treatment, with or without sclerotherapy or surgery.

Regarding the complication rates, no significant differences were found between the sclerosing agents nor the different morphological subtypes. Bleomycin is currently one of the most frequently used sclerosing agents worldwide, and complications rates in literature range from 0 to 20% [6, 12, 19, 29–34]. The most common complications reported are infections, hematomas, and hyperpigmentation [6, 31, 32, 34, 35], which is in accordance with our results (Table 3).

A major concern in the use of bleomycin is the risk of pulmonary toxicity, which is shown to be a rare and dose-related risk [36]. In this study, being one of the largest cohorts studied, no known cases of pulmonary toxicity were identified. Thus, the risk of bleomycin-induced pulmonary toxicity seems to be negligible when used in dosages documented in our protocol. The patient who developed a pneumonia after bleomycin injection had a bacterial pneumonia and furthermore a high risk a priori because of a tracheal cannula in situ. Currently, no standard follow-up protocol is used in our hospital to quantify pulmonary function after use of bleomycin. Whether the addition of pulmonary function tests and/or radiographic assessment to current standard care is useful and cost-effective in the detection of pulmonary complications after use of bleomycin has not yet been clarified.

Regarding the other agents used in our center, complication rates were similar to those reported in other studies [5, 21, 22, 37–42]. The complication rate of lauromacrogol ranged from 0 to 24% in literature, including infections (3%) and hematomas (13–24%) [21, 37]. Our population had a complication rate of 12% without infections and fewer cases of hematomas (5%). Complication rates after doxycycline injection ranged from 0 to 14% in literature, including infection and hemorrhage [5, 22, 38–40], which was similar in our study population (13%). Ethanol was only used in a few exceptional cases, and no complications were identified, which is in accordance to known studies [41, 42].

This study has some limitations inherent to the retrospective study design, in which randomization of the agent was not performed. Considerations on which agent to be used in specific cases were based on expert opinion, since no international guidelines are currently available. Unfortunately, the reason of this choice was not always well documented. The size of the study is a strength in comparison with other studies, but still the reliability of statistical analyses is limited for smaller subgroups. The response rate was determined based on clinical descriptions in the patients’ records, which is subject to the perception of the physician. This may influence the reliability of data, but it probably does not compromise the ability to assess for trends. Future studies are recommended to investigate which approach is best to measure and quantify response after sclerotherapy of (V)LMs, for example, patient-reported outcomes, physical examination, or post-procedural imaging. In these studies, it could be beneficial to use the updated SIR classification, if this is validated properly for a widespread use [43]. Patient-related outcomes are particularly important to focus on in future studies, as proposed by the OVAMA project [44]. In this consensus study, a core outcome set was developed for clinical research on peripheral vascular malformations, and international consensus was reached that patient-reported outcomes play a significant role. These include the item pain and it would be interesting to investigate the differences in intensity and duration of pain between various sclerosing agents. Overall, prospective studies are needed to strengthen the results to determine the most potent sclerosing agent in each subtype and to develop guidelines for patient- or lesion-specific care.

## Conclusions

Complication rates after sclerotherapy of (veno)lymphatic malformations in children are low and mostly include minor complications. No significant differences were found in complication rates between the different sclerosing agents. All agents are effective; however, bleomycin and a combination of agents seem to be most effective in the treatment of pure lymphatic malformations. In clinical care, special attention should be paid to mixed macrocystic and microcystic (veno)lymphatic malformations, because the results of this study indicate that these lesions are more difficult to treat effectively compared with lesions with either one of these components.

## Abbreviations

LMs: Lymphatic malformations
VLMs: Venolymphatic malformations
